# Exploring how organizational virtuousness shapes nurses’ occupational commitment: the mediating role of just culture

**DOI:** 10.1186/s12912-025-03835-x

**Published:** 2025-09-10

**Authors:** Amal Diab Ghanem Atalla, Samia Roshdy Soliman Osman, Heba Ahmed Mohsen Hassen, Faygah Shibily, Ohood Felemban, Makiah Mohammed Shebaili, Wafaa Hassan Mostafa, Aziza Ibrahim Abd El Kader Mohamed, Hoda Sayed Mohamed Sayed, Maaly Zayed Mohammad, Samia Mohamed Sobhi Mohamed

**Affiliations:** 1 Department of Nursing Administration, Faculty of Nursing, Alexandria University, Alexandria, Egypt; 2 Department of Nursing Administration, Faculty of Nursing, Damanhour University, El Behira, Egypt; 3 Department of Nursing Administration, Faculty of Nursing, Rashid University, El Behira, Egypt; 4 Department of Gerontological Nursing, Faculty of Nursing, University of Alexandria, Alexandria, Egypt; 5 Critical Care Nursing Department, Faculty of Nursing, King Abdulaziz University, Jeddah, Saudi Arabia; 6 Public Health Nursing Department, Faculty of Nursing, King Abdulaziz University, Jeddah, Saudi Arabia; 7 Department of Medical Surgical Nursing, Al Ghad College for Applied Medical Sciences, Jeddah, Saudi Arabia; 8 Department of Medical Surgical Nursing, Faculty of Nursing, Cairo University, Cairo, Egypt; 9 Department of Family and Community Health Nursing, Faculty of Nursing, Badr University in Cairo, Cairo, Egypt; 10 Department of Nursing Administration, Faculty of Nursing, Cairo University, Cairo, Egypt

**Keywords:** Organizational virtuousness, Just culture, Occupational commitment, Nursing workforce, Structural equation modeling, Healthcare organizations

## Abstract

**Background:**

Organizational virtuousness and just culture, which both foster justice, honesty, and trust, have a major impact on positive work environments in the healthcare industry. Strengthening nurses’ emotional engagement and vocational commitment requires these components. With an emphasis on the mediating function of just culture, this study attempts to investigate the relationship between organizational virtuousness and nurses’ vocational commitment.

**Methods:**

This study used a descriptive correlational design that was informed by the STROBE checklist. A non-probability convenience sample from Alexandria Main University Hospital was chosen following accepted structural equation modeling (SEM) principles to guarantee sufficient statistical power and trustworthy parameter estimation. A sample of 400 nurses was considered adequate due to the model’s moderate complexity, the use of 61-item measurement methods, and the inclusion of several latent variables. Three validated tools—the Just Culture Survey, the Occupational Commitment Survey, and the Organizational Virtuousness Scale—were used to gather data.

**Results:**

According to linear regression analysis, nurses’ occupational commitment was significantly predicted by both organizational virtuousness and just culture, which together accounted for 15.5% of the variation. Organizational virtuousness was a significant positive predictor (B = 0.220), meaning that occupational commitment rose by 0.220 units for every unit rise in perceived virtuousness. The greatest predictor was only culture (B = 0.352, β = 0.342, t = 7.207, *p* < 0.001, 95% CI [0.256, 0.448]), underscoring its crucial influence on commitment. These results highlight the value of open, equitable workplace cultures and moral organizational climates in raising nurses’ levels of professional engagement. These correlations were further validated by the structural equation model, which showed good model fit (GFI = 0.961, AGFI = 0.941, RMSEA = 0.069).

**Conclusions:**

Nurses’ commitment to their work is strengthened by an organization’s moral character and fair culture. These elements improve trust, engagement, and emotional involvement by creating a courteous, moral, and encouraging work atmosphere. In healthcare contexts, fostering such cultural values improves employee retention, productivity, and overall organizational resilience.

**Clinical trial number:**

Not applicable.

**Supplementary Information:**

The online version contains supplementary material available at 10.1186/s12912-025-03835-x.

## Literature review

In today’s healthcare systems, where complexity, unpredictability, and emotional labor are routine, nursing professionals often operate under intense pressure. Globally, nurses are the foundation of healthcare systems, and their unwavering dedication to their jobs is essential to upholding high patient care standards, lowering attrition, and improving organizational stability [1]. However, burnout and diminished commitment among nurses are frequently caused by growing job stress, ethical dilemmas, and organizational inefficiencies [2]. To address these issues, current studies have shifted to positive organizational scholarship to comprehend how particular organizational traits might promote commitment, resilience, and employee engagement [3, 4]. One such new concept is organizational virtuousness, which is the collective expression of virtues like forgiveness, compassion, integrity, optimism, and trust within an organization [4].

Organizational virtuousness has been demonstrated to affect several favorable staff nurses’ outcomes, such as psychological well-being, job satisfaction, and engagement, but little is known about how it relates to occupational commitment, particularly in high-stress occupations like nursing [5]. Furthermore, how organizational virtuousness influences this commitment is not well understood. A key mediating element in this relationship would be the idea of a just culture, which places an emphasis on growing from mistakes rather than placing blame. Just cultures encourage psychological safety and accountability, which may strengthen the beneficial effects of moral workplace cultures on nurses’ commitment to their work [6].

### Organizational virtuousness

Organizational virtuousness is a concept that was created within the framework of positive organizational study and describes the collective execution of virtues that elevate both individuals and organizations. It comprises five interrelated virtues: optimism, trust, compassion, integrity, and forgiveness [7]. All the virtues help create a morally elevating workplace where people feel appreciated and motivated to put the benefit of the group ahead of their own. Optimism reflects the organization’s belief in its ability to overcome adversity, encouraging hope and resilience among staff. Trust is foundational to cooperation and psychological safety, particularly in multidisciplinary healthcare teams. Compassion is essential in emotionally laborious settings like nursing, where mutual care mitigates burnout. Integrity reflects the moral backbone of the organization and enhances alignment between personal and organizational values. Forgiveness facilitates recovery from mistakes and fosters a culture of growth rather than fear [7, 8].

A virtuous organization is more likely to be predictable by promoting tolerance among members and promoting regular connections with the organization [or its leaders] and other members. This regular contact creates a feeling of closeness and unity by facilitating the sharing of knowledge and feedback. Furthermore, it strengthens nurses’ corporate identities by assisting them in comprehending the distinctions between “insiders” and “outsiders” within the company [9].

Strong relationships among coworkers are developed when nurses have a positive perception of their hospital and are drawn to its moral staff. These social connections satisfy nurses’ needs for safety and social contact, which increases their level of satisfaction. People’s thought-action repertoires are expanded by positive emotions from working in a moral organization, which enables them to think creatively and overcome obstacles. Employee annoyance in managing daily workplace challenges is reduced by this readiness to cooperate constructively to solve problems [5]. As a result, individuals adopt constructive actions that enhance their sense of belonging to the company, which promotes goodwill, greater loyalty, trust, and occupational commitment among nurses [10].

### Occupational commitment among nurses

The concept of “occupational commitment” refers to a person’s psychological bond with their line of work. This type of dedication is especially crucial for nurses because of the emotionally taxing and morally challenging nature of their employment [11]. Occupational commitment has been linked to improved patient outcomes, higher job satisfaction, and lower inclinations to leave the company. Finding organizational components that might encourage or increase nurses’ commitment is crucial because the high-stress nature of the healthcare sector frequently makes it difficult for them to remain dedicated [12]. Three dimensions make up occupational commitment and are operationalized as follows: Affective commitment: Emotional attachment and identification with the profession. Normative commitment: A sense of obligation to remain in the profession. Continuance commitment: divided within the subgroups of accumulated costs and limited alternatives [13, 14].

Strong occupational commitment in nursing is linked to better patient outcomes, increased resilience, and less turnover [15]. By addressing alternative costs, emotional investment, and value congruence, Sharbaugh’s 24-item measure effectively captures this complexity [14]. A more accurate understanding of how various organizational variables, such as virtue and justice, influence distinct aspects of professional commitment is made possible by this multidimensional paradigm. However, the function of a positive organizational culture, particularly one that is founded on moral values, has not been thoroughly investigated. Nurses may internalize these principles and become more emotionally invested in their work if they believe their organization to be moral, encouraging, and caring [16].

### Just culture

Within healthcare organizations, a just culture fosters accountability, learning, and openness. It promotes trust and psychological safety by allowing people to confess mistakes without worrying about the consequences. A Just Culture denotes a systems-thinking approach to error management and organizational learning. Rather than placing blame only on individuals, it emphasizes systemic understanding, psychological safety, and ethical accountability [17]. The just culture framework used in this study includes five components: Feedback and communication, Openness of communication with supervisors, Balance between accountability and blame, Quality of the event reporting process, and Commitment to continuous improvement [18].

A just culture in healthcare is crucial for maintaining employee morale and ensuring patient safety. It has been demonstrated to improve organizational learning, foster open communication, and improve team performance [17]. Crucially, by offering a tangible framework wherein virtues like forgiveness and compassion are operationalized through equitable and constructive reactions to mistakes, a just culture can also amplify the effects of organizational virtue. Because of this, it is a believable mediator between organizational virtuousness and occupational commitment [19].

Previous studies have examined professional commitment, just culture, and organizational virtue separately. The impact of organizational virtuousness [OV] on job burnout and work engagement was examined, with a focus on the moderating role of organizational support. The study also explores how work engagement is impacted by job burnout [19]. The findings showed a positive association between work engagement and organizational virtuousness and a negative correlation between OV and job weariness. Additionally, the results validated the negative correlation between job burnout and work engagement. Additionally, it was discovered that the association between the variables in the research model is not moderated by organizational support. These findings have useful implications for how hotels and travel companies might support their employees in the workplace [19].

Another study conducted investigates the relationship between occupational stress and organizational commitment among staff nurses [20]. The study revealed that significant statistical correlations were found between research participants’ attributes and their levels of overall organizational commitment and occupational stress. The study participants’ occupational stress and their overall organizational commitment showed a statistically significant, somewhat unfavorable association. The study examines the moderating effect of just culture on the association between oncology nurses’ silent actions regarding patient safety and the nursing practice environment [21]. The study’s conclusions showed a strong unfavorable association between the practice environment as a whole and nurses’ silence regarding patient safety. This association is strengthened by the interaction between the nurse practice environment and just culture, which improves error reporting [21].

Currently, current literature lacks strong evidence on how and through what mechanisms positive organizational environments translate into professional commitment, especially in emotionally demanding fields such as nursing. This study aims to tie this gap by testing the mediating role of just culture in the relationship between organizational virtuousness and nurses’ occupational commitment. Thus, this study aims to investigate the relationship between organizational virtuousness and nurses’ occupational commitment, with a particular emphasis on the mediating function of just culture. This allows it to integrate ideas from nursing management, organizational justice, and positive organizational studies, contributing to both theory and practice. To build resilient, dedicated, and high-performing nursing teams in the face of ever-increasing obstacles, healthcare executives can benefit greatly from an understanding of how favorable ethical climates influence professional attitudes.

### Theoretical underpinning: social exchange and organizational justice

The proposed model is grounded in Social Exchange Theory [SET] [22], which asserts that relationships are formed and maintained based on reciprocal exchanges of value. In this context, when nurses perceive that their organization upholds virtuous values and provides a just and respectful culture, they are likely to reciprocate with increased loyalty and professional commitment [23]. Furthermore, Organizational Justice Theory [24] complements this view by emphasizing the importance of fairness and ethical treatment in shaping employee attitudes and behaviors. Thus, the mediating role of just culture is consistent with both theoretical frameworks. It represents the cognitive and affective processing of organizational behavior that informs whether nurses feel morally supported and professionally respected — key precursors to occupational commitment [25].

When combined, these two ideas offer a strong framework for comprehending how organizational contexts affect nurses’ dedication to their jobs. According to the Social Exchange Theory, employees use perceived investments and advantages to gauge how well they get along with the company. Nurses perceive organizational virtues like integrity, compassion, and respect as indications of the organization’s commitment to their welfare, which in turn inspires a sense of duty and dedication. According to Organizational Justice Theory, people’s perceptions of the legitimacy of authority and their place in the system are influenced by fair procedures, open communication, and balanced accountability, all of which are essential elements of a just culture. Nurses who believe that their workplace is just and morally aligned are more likely to build psychological safety and trust, which strengthens their emotional bond and commitment to the profession. Therefore, fair culture acts as a psychological mechanism as well as a mediator, allowing the reciprocal and justice-based demands of the workplace to be internalized and converted into long-term occupational commitment.

### Conceptual model of the study

**Fig. 1:**
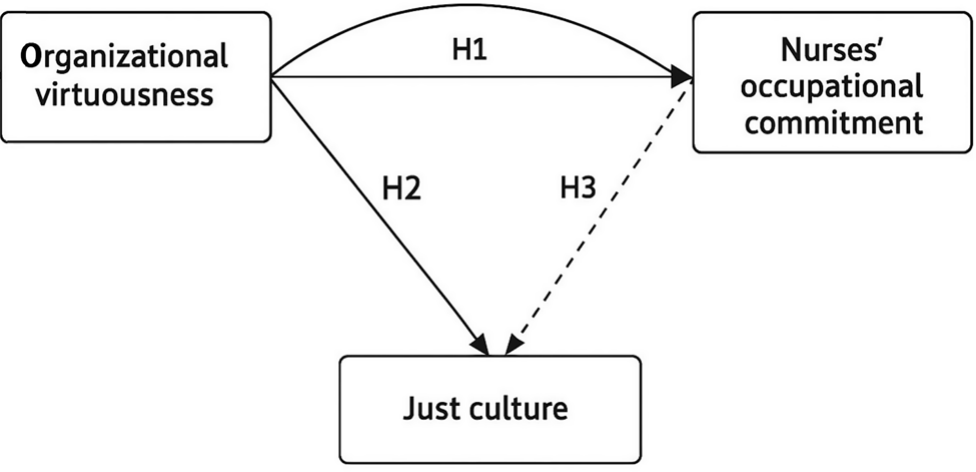
Conceptual framework illustrating the mediating role of just culture in the relationship between organizational virtuousness and nurses’ occupational commitment

The researchers proposed a conceptual model Fig. 1 for this study based on the earlier conceptualization. Just culture acts as a mediator between the independent variable of organizational virtue and the dependent variable of nurses’ occupational commitment. The following theories are put forth:

#### H1

Organizational virtuousness is positively associated with nurses’ occupational commitment.

#### H2

Organizational virtuousness is positively associated with a just culture.

#### H3

Just culture is positively associated with nurses’ occupational commitment.

#### H4

Just culture mediates the relationship between organizational virtuousness and nurses’ occupational commitment.

### Significance of the study

Organizational virtuousness, encompassing attributes such as trust, compassion, integrity, forgiveness, and optimism, has been identified as a pivotal factor in enhancing employees’ professional commitment [26]. In the nursing profession, where ethical standards and emotional resilience are paramount, the presence of organizational virtuousness can significantly influence nurses’ commitment to their roles [27]. Recent studies have demonstrated that a positive ethical climate within healthcare organizations correlates with increased job satisfaction and organizational commitment among nurses [28]. Furthermore, adherence to organizational ethics has been linked to reduced job burnout and heightened organizational commitment, underscoring the importance of virtuous organizational practices in fostering a committed nursing workforce [29]. A just culture fosters an environment where nurses feel safe to report errors and engage in open communication, thereby reinforcing trust and integrity within the organization. Empirical evidence suggests that the implementation of a just culture positively influences nurses’ perceptions of fairness and support, leading to enhanced professional commitment [29, 30].

Moreover, organizations that prioritize just culture principles have observed improvements in patient safety activities and a reduction in adverse events, further highlighting the role of just culture in promoting a committed and effective nursing workforce [30]. Integrating the concepts of organizational virtuousness and just culture provides a comprehensive framework for understanding and enhancing nurses’ occupational commitment. Organizational virtuousness lays the foundation for ethical and supportive workplace practices, while a just culture operationalizes these virtues through fair and transparent policies [31]. Together, they create an environment that not only values ethical behavior but also actively supports it through systemic structures. This synergistic relationship has been shown to mitigate work-related stress and burnout among nurses, leading to increased job satisfaction and retention. By fostering both virtuous organizational values and a just culture, healthcare institutions can effectively enhance nurses’ commitment to their profession [32].

### Study design

A descriptive correlational cross-sectional study design was employed in an Egyptian hospital, adhering to the STROBE (Strengthening the Reporting of Observational studies in Epidemiology) guidelines. A descriptive correlational cross-sectional design was chosen as it allows examination of the relationships among organizational virtuousness, just culture, and occupational commitment at a single point in time, providing empirical insights into their interconnections without manipulating variables.

### Setting

This study was conducted at Alexandria Main University Hospital, which is affiliated with Alexandria University. It was chosen as the study location because of its special qualities that closely match the goals of this study. With more than 6,760 beds and 23 critical care units, it is Alexandria’s largest educational university hospital. It offers a wide range of healthcare services, making it the perfect place to study workforce dynamics and organizational culture.

To investigate organizational virtue and just culture, the hospital’s relationship with Alexandria University guarantees the existence of organized administrative processes, continual professional growth, and exposure to academic and clinical best practices. Additionally, because it is a public, non-profit healthcare facility, it provides a realistic setting for researching the occupational commitment of nurses who deal with a variety of patient requirements, intense pressure, and limited resources.

This environment is ideal for examining how fairness and positive organizational values can affect nurses’ dedication to their work, particularly in critical care units where psychological safety, team cohesiveness, and moral decision-making are crucial. The study’s conclusions from this context might apply to comparable healthcare settings with low and middle incomes that want to promote long-term employee engagement.

### Sampling

To ensure sufficient statistical power and accurate parameter estimation in structural equation modeling (SEM), the non-probability convenience sample size was selected in compliance with established guidelines in the literature. According to Molwus et al. (2013), 200–400 people are considered an acceptable sample size for models of moderate complexity [33]. Since the current study used SEM with a model that comprised many latent variables and a measurement tool with 61 questions, a sample of 400 nurses was deemed sufficient. This sample size falls within the recommended range and provides a strong basis for evaluating the suggested relationships between the constructs under study. Participants had to meet the following criteria to be included: (1) willing to participate voluntarily in the research after providing informed consent. (2) possessing at least six months of professional nursing experience.

### Study tools

Three tools were used in this study as follows:

#### Tool 1: organizational virtuousness scale (OVS)

Cameron et al. created this tool (2004). This tool has fifteen items and four dimensions in total. OVS has three items for optimism, three items for trust, three items for compassion, three items for integrity, and three items for forgiveness [7]. A 5-point Likert scale, from strongly agree (5) to strongly disagree (1), was used to score the responses. The total score falls between 15 and 75. Organizational Virtuousness ranges from 15 to 35 for low levels, 35 to 55 for moderate levels, and 55 to 75 for high levels. A high degree of organizational virtuousness is indicated by higher scores.

#### Tool 2: nurses’ occupational commitment survey

Based on the work of Meyer et al. (1993) [13], the Occupational Commitment Survey, a 24-item Likert scale, was adapted by Blau (2003) [14]. It measures four dimensions of occupational commitment: affective (items 1–6), normative (items 7–12), continuance–accumulated costs (items 13–20), and continuance–alternatives (items 21–24). Although the original items are grouped by subscale, they were randomized for this study. Subscale scores were calculated by summing their respective items. OCS’s dependability was assessed using Cronbach’s alpha.84 was the alpha score [13]. A 6-point Likert scale, with 1 denoting “strongly disagree” and 6 denoting “strongly agree,” is used in the Occupational Commitment Survey. The total score falls between 24 and 144. Occupational commitment ranges from 24 to 64 for low levels, 64 to 104 for moderate levels, and 104 to 144 for high levels. Higher ratings show that nurses are very committed to their jobs.

#### Tool 3: just culture assessment tool survey (JCATS)

The JCATS was developed by Petschonek et al. (2013) to measure the just culture perceptions of healthcare professionals in the hospital setting. The questions of the JCATS survey measured the staff nurses’ safety culture perceptions in the dimensions of balance and trust, openness of communication, quality of the event-reporting process, feedback and communication about events, and an overall goal of continuous improvement. used the 22 questions that corresponded to the five domains to measure the overall just culture scores [18]. The tool uses a 7-point Likert scale for the responses, ranging from 1 (strongly disagree) to 7 (strongly agree). The Cronbach’s alpha was found to be higher than 0.70 in all dimensions. The overall score ranges from 22 to 154. Low levels of just culture range from 22˂66, moderate levels range from 66˂110, and high levels range from 110 to 154. Higher scores indicate a high level of just culture among nurses.

In addition, a demographic datasheet was developed by the researcher; it included questions related to gender, age, educational qualification, working unit, years of service, and nursing experience.

### Tools validity

A thorough process of translation and validation was carried out to guarantee the validity and cultural relevance of the study tools, which are the Occupational Commitment Questionnaire, the Just Culture Assessment Tool, and the Organizational Virtuousness Scale. First, bilingual professionals with experience in organizational research and healthcare translated each tool from English into Arabic. To find discrepancies and guarantee semantic accuracy, a different team of multilingual experts then back-translated into English. A panel of five subject area experts then assessed the reconciled Arabic versions to determine their clarity, cultural appropriateness, and content relevance in the context of Egyptian healthcare. Confirmatory Factor Analysis (CFA) was used to confirm construct validity.

With values above the acceptable cutoff of 0.60, the Kaiser-Meyer-Olkin (KMO) measure verified sampling adequacy for all tools: 0.921 for the Organizational Virtuousness Scale, 0.913 for the Just Culture tool, and 0.894 for the Occupational Commitment scale, all with p-values < 0.001. All items had factor loadings greater than 0.70, demonstrating strong internal consistency. While discriminant validity was verified by making sure that each construct’s AVE was greater than the squared correlations with other constructs, convergent validity was supported by Average Variance Extracted (AVE) values over 0.50 across all constructs. These results confirm that the study’s tools are suitable for evaluating the desired variables in the context of Egyptian nursing and have good psychometric quality.

### Ethical considerations

The Alexandria University Research Ethics Committee of the College of Nursing approved the study process, and the research serial number is AU-20-8-347 (IRB00013620(9/19/2025). The purpose of the study was to explain to nurses before their consent, and each questionnaire was given a code number to protect their identity and privacy. The researchers were assured that the data would be used exclusively for research purposes and were also offered the option to withdraw from the study.

The study ensured participant anonymity and confidentiality using several measures. Nurses’ IDs were eliminated before data processing, and responses were coded using participant ID numbers rather than names. All participants provided their informed consent after being fully informed about the study’s purpose and their right to discontinue participation at any time without facing any repercussions. Paper-based records were stored in a locked cabinet, while electronic files were password-protected to prevent unauthorized access. By following ethical criteria, such as the Declaration of Helsinki, participant data were treated ethically throughout the research process, and privacy was preserved.

### Pilot study and reliability

The pilot project was approved by 10% of the nurses (*n* = 40) to ensure the tools’ usability and utility and to identify any potential issues or roadblocks during data collection. Nothing needed to be changed. To prevent data contamination, participants in the pilot experiment were not allowed to continue the study. The results demonstrated that no adjustments were necessary because each item was understandable, relevant, and consistent with the study’s objectives. The participants reported that they had no doubts or trouble understanding the questions.

The dependability Cronbach’s alpha values for the pilot study mirrored those of the complete study, confirming strong internal consistency. The sufficient range in response patterns and the absence of floor or ceiling effects demonstrate that the scales were successful in capturing the intended components. These outcomes demonstrated that the apparatus was suitable for gathering substantial volumes of data.

### Overcame the problem of common method biases

To combat common method bias (CMB), the authors combined statistical, procedural, and design controls. They ensure participant anonymity and confidentiality to reduce social desirability bias and encourage honest answers. They also used a range of response styles, provided clear instructions, and separated the questionnaire into multiple measures to lessen typical response patterns. Harman’s single-factor test supported the result that no single factor accounted for most of the variance, suggesting that CMB was not a significant concern. Furthermore, confirmatory factor analysis (CFA), which ensures distinct and uncorrelated components, was used to validate the measurement model.

To increase clarity and eliminate any ambiguities that can lead to CMB, the authors additionally conducted pilot research with 10% of the sample. They were able to minimize any potential CMB impacts in the study by combining these methods.

### Data collection

Every participant received a copy of the study questionnaires. Each nurse received a questionnaire from the researchers by hand, and they gathered the filled-out forms. Each nurse had two minutes to explain the purpose of the study before being asked to return it to the researcher. To guarantee the respondents’ objectivity, the integrity of their ideas, and the completion of all questions, these scales were filled out in front of the researcher. It was simple to keep an eye on the delivery and collect data to ensure the best response rate because they were linked to specific working units. Completing the questions should take fifteen to twenty minutes. Two months, from April 2025 to June 2025, were used to collect the data. Every query from the nurses was answered, and explanations were given.

### Data analysis

A systematic coding and entry procedure was used to guarantee the integrity and accuracy of the data. IBM SPSS AMOS (Version 23) and IBM SPSS Statistics (Version 23) were used to enter and analyze the data. Data cleaning techniques, such as checks for missing values, suitable imputation, and outlier analysis, were used to improve data quality and lessen bias after responses were double-checked for accuracy.

The demographics of the participants and the main research variables: organizational virtuousness, just culture, and professional commitment, were described using descriptive statistics like frequencies, percentages, means, and standard deviations. One-way ANOVA and independent-sample t-tests were used to compare groups according to demographic traits.

Pearson’s correlation was used to look at the relationships between the main variables. Cronbach’s alpha and composite reliability (CR) validated the instruments’ internal consistency and reliability, while regression analysis was used to evaluate direct effects between variables. Construct validity was evaluated using Confirmatory Factor Analysis (CFA). A Structural Equation Model (SEM) was used to examine the mediating function of simple culture. The direction and strength of correlations were ascertained using path coefficients. The product of route coefficients was used to evaluate the indirect impact of organizational virtue on occupational commitment through just culture. Typical indices were used to assess the model fit: **Chi-square/df** (values < 3 indicate good fit), **Comparative Fit Index (CFI)**, and **Incremental Fit Index (IFI)** (values ≥ 0.90 suggest excellent fit), and Root **Mean Square Error of Approximation (RMSEA)** (values ≤ 0.08 indicate acceptable approximation).

## Results

Table 1 shows that the largest group of participants (43.3%) was between the ages of 40 and 49, with an average age of 41.9 years (SD = 7.8). Nearly half (47.8%) were married, and the majority (77.3%) were female. Regarding credentials, 23.0% had a Bachelor of Nursing, 45.3% had a nursing high school certificate, and 43.8% had an institution diploma. Two-thirds (66.8%) worked in internal units, and almost one-third worked in acute care units. With an average of 14.1 years of nursing experience and 13.7 years in their present role, the majority (64.0%) had at least 15 years of experience (Table 1).

**Table 1:** Distribution of the studied nurses according to demographic data (*n* = 400)

Demographic characteristics	No.	%
**Age (years)**		
20-<30	125	31.3
30-<40	40	10.0
40-<50	173	43.3
≥ 50	62	15.5
Mean ± SD	41.9 ± 7.80	
**Sex**		
Male	91	22.8
Female	309	77.3
**Marital status**		
Married	191	47.8
Single	80	20.0
Divorced	57	14.3
Widow	72	18.0
**Level of education**		
Bachelor of nursing	92	23.0
Nursing high school diploma	181	45.3
Nursing institute diploma	127	31.8
**Unit**		
Internal	267	66.8
ICU	133	33.3
**Years of experience in the nursing profession**		
1 - <5	21	5.3
5-<10	103	25.8
10-<15	20	5.0
≥ 15	256	64.0
Mean ± SD	14.1 ± 6.2	
**Years of experience in the job position**		
1 - <5	28	7.0
5-<10	96	24.0
10-<15	31	7.8
≥ 15	245	61.3
Mean ± SD	13.7 ± 5.9	

As evident in Tables 2 and 71.0% of the 400 nurses who were surveyed had moderate-to-high organizational virtuousness (M = 51.37, SD = 13.74; 60.62% ± 22.90). The subscales with the greatest scores were forgiveness (65.02%), optimism (64.79%), compassion (59.79%), trust (59.71%), and integrity (53.79%). With 71.8% in the high category (M = 106.68, SD = 23.10; 68.90% ± 19.25), occupational commitment was likewise strong. Normative (70.50%), continuation (68.02%), alternative cost (67.17%), and emotional commitment (69.07%) were the subscale scores (Table 2).

**Table 2:** Distribution of the studied nurses according to their levels and mean percent score of organizational virtuousness, occupational commitment, and just culture (*n* = 400)

	Low		Moderate		High		Total score	Mean score	Mean percent score
	No.	%	No.	%	No.	%	Mean ± SD	Mean ± SD	Mean ± SD
**Organizational Virtuousness**	44	11.0	72	18.0	284	71.0	51.37 ± 13.74	3.42 ± 0.92	60.62 ± 22.90
Optimism	50	12.5	69	17.3	281	70.3	10.78 ± 2.96	3.59 ± 0.99	64.79 ± 24.66
Trust	53	13.3	71	17.8	276	69.0	10.17 ± 3.48	3.39 ± 1.16	59.71 ± 29.02
Compassion	41	10.3	83	20.8	276	69.0	10.18 ± 3.22	3.41 ± 1.07	59.79 ± 26.81
Integrity	49	12.3	65	16.3	286	71.5	9.45 ± 3.31	3.15 ± 1.10	53.79 ± 27.62
Forgiveness	54	13.5	64	16.0	282	70.5	10.80 ± 2.98	3.60 ± 0.99	65.02 ± 24.87
**Occupational Commitment**	**13**	**3.3**	**100**	**25.0**	**287**	**71.8**	**106.68 ± 23.10**	**4.45 ± 0.96**	**68.90 ± 19.25**
Affective occupational commitment	25	6.3	105	26.3	270	67.5	26.72 ± 6.69	4.45 ± 1.11	69.07 ± 22.29
Normative occupational commitment	14	3.5	106	26.5	280	70.0	27.15 ± 6.94	4.52 ± 1.16	70.50 ± 23.14
**Continuance commitment**	**4**	**1.0**	**102**	**25.5**	**294**	**73.5**	**52.81 ± 12.56**	**4.40 ± 1.05**	**68.02 ± 20.91**
* Alternative Cost*	7	1.8	102	25.5	291	72.8	34.87 ± 9.74	4.36 ± 1.22	67.17 ± 24.33
* Alternatives*	15	3.8	110	27.5	275	68.8	17.94 ± 3.84	4.49 ± 0.96	69.70 ± 19.21
**Just Culture**	**12**	**3.0**	**49**	**12.3**	**339**	**84.8**	**97.89 ± 22.44**	**4.90 ± 1.12**	**64.93 ± 18.68**
Feedback and Communication	11	2.8	54	13.5	335	83.8	14.32 ± 5.15	4.78 ± 1.72	62.96 ± 28.59
Openness of Communication	8	2.0	53	13.3	339	84.8	25.38 ± 7.18	5.08 ± 1.44	67.92 ± 23.92
Balance	12	3.0	64	16.0	324	81.0	24.61 ± 6.26	4.92 ± 1.25	65.37 ± 20.87
Quality of event reporting process	16	4.0	37	9.3	347	86.8	23.04 ± 6.38	4.61 ± 1.27	60.21 ± 21.23
Continuous Improvement	17	4.3	41	10.3	342	85.5	10.54 ± 2.43	5.27 ± 1.21	71.17 ± 20.21

The mean score on the alternatives scale was 17.94 (SD = 3.84; 69.70% ± 19.21%). 84.8% of nurses gave culture a high rating overall, with a mean score of 97.89 (SD = 22.44; 64.93% ± 18.68%). The continuous improvement subscale had the highest scores (71.17%), followed by the event-reporting procedure (60.21%), feedback and communication (62.96%), balance (65.37%), and openness of communication (67.92%).

Relationships between Organizational Virtuousness (F1), Just Culture (F3), and Occupational Commitment (F2) were investigated using the structural equation model. Optimism, trust, compassion, honesty, and forgiveness were used to measure organizational virtue; balance, open communication, feedback, event reporting, and continuous improvement were used to measure just culture; and affective, normative, alternative, and alternative costs were used to measure occupational commitment. Construct validity was validated by strong standardized loadings. The mediation effect of Just Culture was substantiated by significant routes (F1→F2 = 5.21, F1→F3 = 2.62, and F3→F2 = 4.17), which demonstrated that Organizational Virtuousness both directly and indirectly increases Occupational Commitment.

**Fig. 2:**
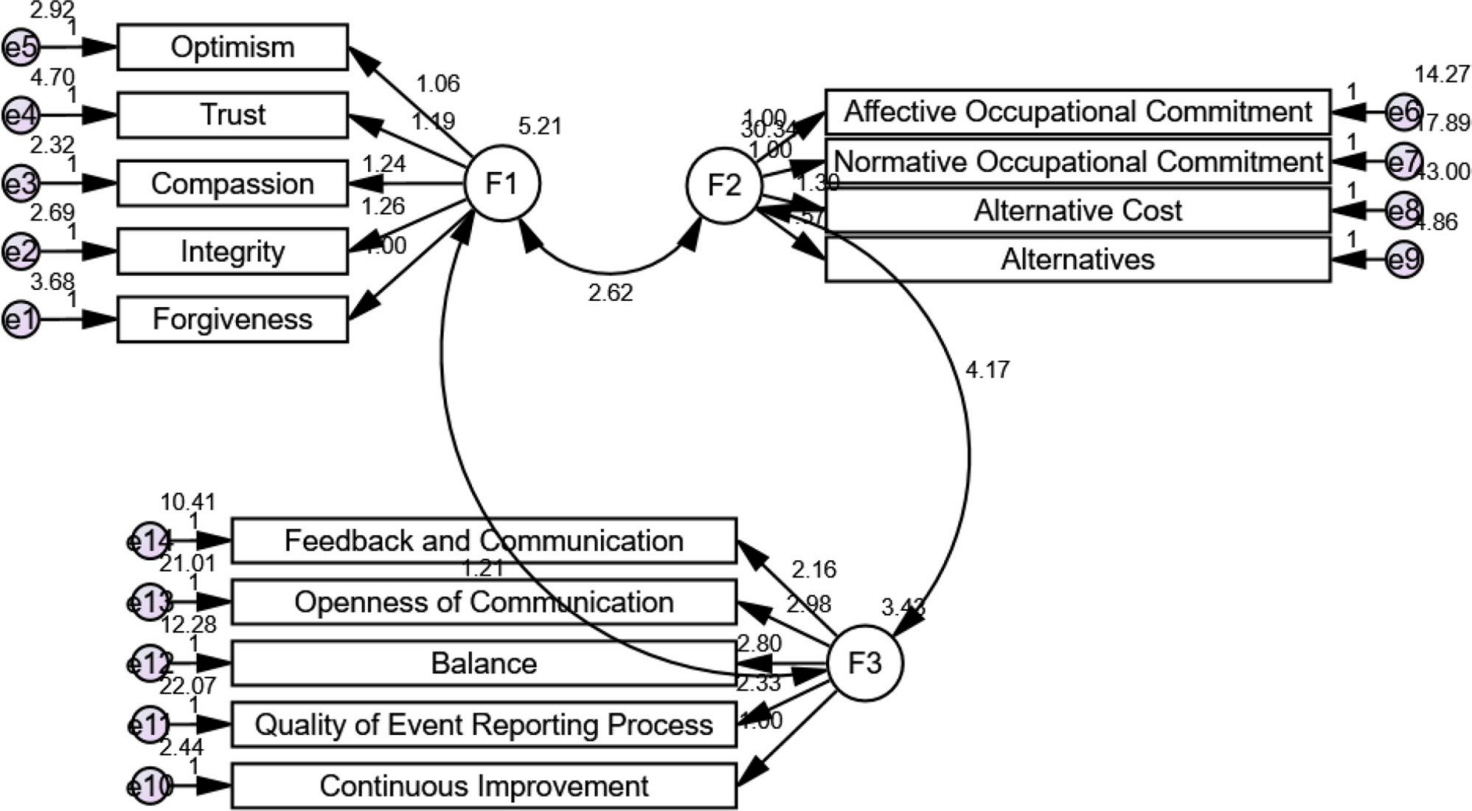
Structural equation model of the relationships among organizational virtuousness, just culture, and occupational commitment F1: organizational virtuousness F2: occupational commitment F3: just culture model fit parameters CFI; IFI; RMSEA (0.928; 0.956; 0.069) odel χ^2^. 2.912 *p* ≤ 0.001. CFI: Comparative Fit Index, IFI: Incremental Fit Index, RMSEA: Root Mean Square Error of Approximation

Table 3 illustrates that the validity of the measurement model was confirmed by Table 3, which demonstrates that all indicator loadings on their corresponding latent constructs were statistically significant (*p* < 0.001). While Openness of Communication and Quality of Event Reporting were important markers of Just Culture, Integrity was most strongly associated with Organizational Virtue. Alternative costs displayed the highest loading for Occupational Commitment, highlighting the importance of professional investment. The robustness of the model was strengthened by high critical ratios (C.R. >9.5). The main premise that Organizational Virtue improves nurses’ Occupational Commitment both directly and indirectly through Just Culture is supported by Fig. 2; Table 3.

**Table 3:** Regression weights for the measurement model of organizational virtuousness, just culture, and occupational commitment

			Estimate	S.E.	C.*R*.	*P*
Optimism	<---	**Organizational Virtuousness**	3.677	0.298	12.351*	< 0.001*
Trust	<---	**Organizational Virtuousness**	2.690	0.271	9.926*	< 0.001*
Compassion	<---	**Organizational Virtuousness**	2.319	0.243	9.558*	< 0.001*
Integrity	<---	**Organizational Virtuousness**	4.703	0.396	11.866*	< 0.001*
Forgiveness	<---	**Organizational Virtuousness**	2.920	0.256	11.427*	< 0.001*
Affective occupational commitment	<---	**Occupational Commitment**	14.270	1.481	9.637*	< 0.001*
Normative occupational commitment	<---	**Occupational Commitment**	17.890	1.691	10.577*	< 0.001*
*Alternative Cost*	<---	**Occupational Commitment**	43.004	3.732	11.523*	< 0.001*
*Alternatives*	<---	**Occupational Commitment**	4.862	0.496	9.803*	
Feedback and Communication	<---	**Just Culture**	2.441	0.216	11.292*	< 0.001*
Openness of Communication	<---	**Just Culture**	22.072	1.775	12.438*	< 0.001*
Balance	<---	**Just Culture**	12.279	1.244	9.869*	< 0.001*
Quality of event reporting process	<---	**Just Culture**	21.006	1.883	11.157*	< 0.001*
Continuous Improvement	<---	**Just Culture**	10.410	0.953	10.924*	< 0.001*

All pairwise correlations between the three higher-order constructs were positive and statistically significant, according to Pearson’s correlation analysis. There was a weak but significant correlation between organizational virtuousness and just culture (*r* = 0.237, *p* < 0.001) and occupational commitment (*r* = 0.212, *p* < 0.001). Similarly, there was a moderately positive association between just culture and occupational commitment (*r* = 0.373, *p* < 0.001), suggesting that nurses who believe their organization is morally upright also report higher levels of dedication and a more robust just-culture atmosphere (Table 4). A detailed correlation between each dimension’s subscales is illustrated in a supplementary Table 1.

**Table 4:** Correlation between total organizational virtuousness, just culture, and occupational commitment among nurses (*n* = 400)

Variable	1. Organizational Virtuousness (Total)	2. Occupational Commitment (Total)	3. Just Culture (Total)
1. Organizational Virtuousness (Total)	1		
2. Occupational Commitment (Total)	0.212*	1	
3. Just Culture (Total)	0.237*	0.373*	1

r: Pearson coefficient *: Statistically significant at *p* ≤ 0.05

As illustrated in Table 5, according to multiple regression analysis, just culture and organizational virtue jointly accounted for 15.5% of the variation in professional commitment (R2 = 0.155, F(2,397) = 36.53, *p* < 0.001). Both were significant predictors, but the stronger predictor was merely culture (B = 0.352, *p* < 0.001) and organizational virtue (B = 0.220, *p* = 0.006). These results suggest that perceptions of a moral organization and an open, equitable culture both have a favorable impact on nurses’ vocational commitment (Table 5).

**Table 5:** Linear regression models for factors affecting occupational commitment (*n* = 400)

Factors	B	Beta	t	*p*	95% CI	
					LL	UL
Organizational Virtuousness	0.220	0.131	2.755*	0.006*	0.063	0.377
Just Culture	0.352	0.342	7.207*	< 0.001*	0.256	0.448

R^2^ = 0.155, Adjusted R^2^ = 0.151, F = 36.532*, *p* < 0.001^*^

F, p: F and p values for the model

R^2^: Coefficient of determination

B: Unstandardized Coefficients

Beta: Standardized Coefficients

t: t-test of significance

CI: Confidence interval

LL: Lower limit

UL: Upper Limit

*: Statistically significant at *p* ≤ 0.05

### Table 6; Fig. 3: direct and indirect effects of organizational virtuousness and just culture on occupational commitment: structural equation modeling results

**Table 6:** Direct and indirect effects of organizational virtuousness and just culture on occupational commitment: structural equation modeling results

Dependent		Independent	Direct effect	Indirect effect	Estimate	Indirect effect	S.E.	C.*R*.	*P*
Just Culture	<---	Organizational Virtuousness	0.388		0.237		0.079	4.881*	< 0.001*
Occupational Commitment	<---	Organizational Virtuousness	0.220	0.137	0.131	0.081	0.080	2.761*	0.006*
Occupational Commitment	<---	Just Culture	0.352		0.342		0.049	7.225*	< 0.001*

Model fit parameters CFI; IFI; RMSEA (1.000; 1.000; 0.072)

Model χ^2^/df. 3.181 *p* ≤ 0.001

CFI: Comparative Fit Index, IFI: Incremental Fit Index, RMSEA: Root Mean Square Error of Approximation

**Fig. 3:**
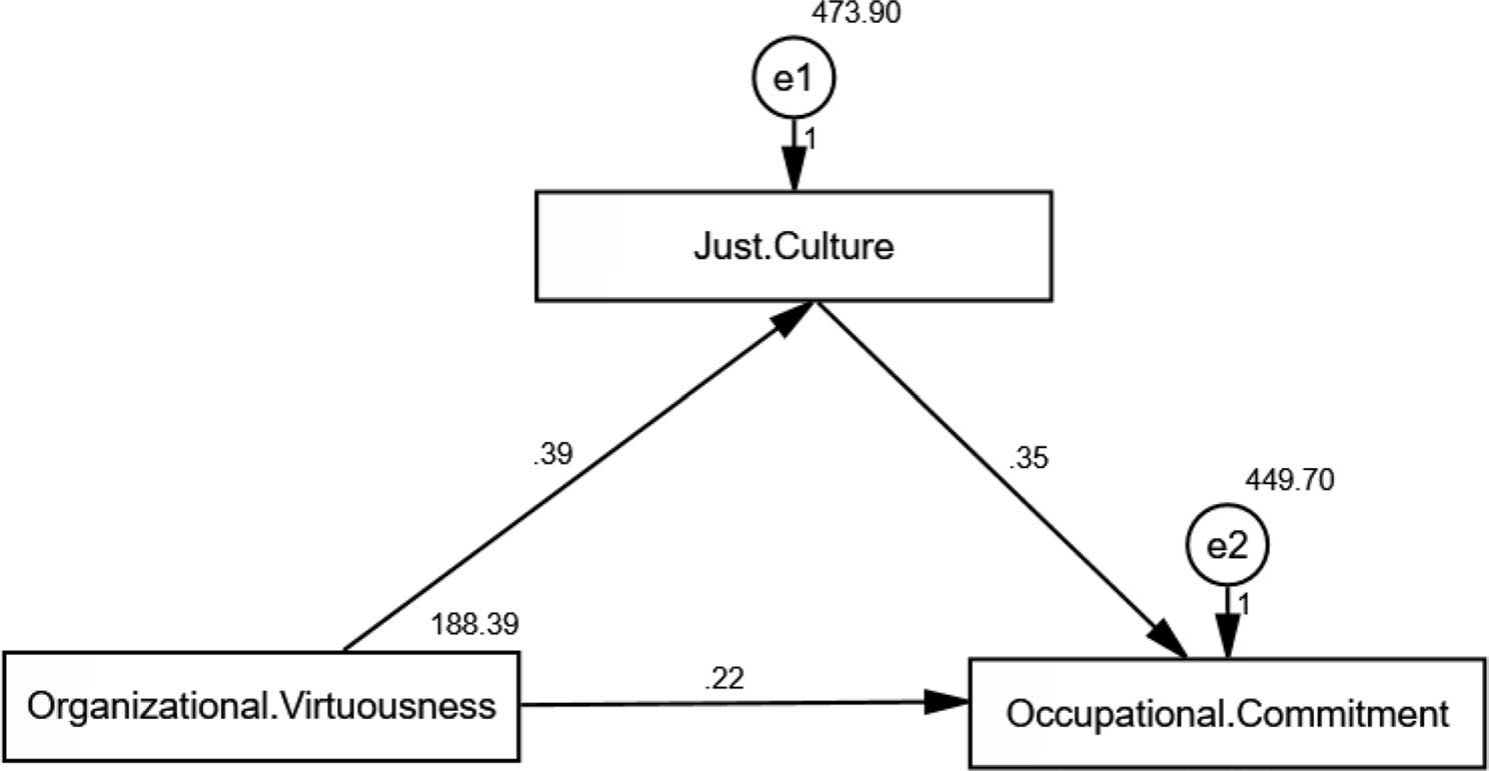
Path analysis of the direct and indirect effect of organizational virtuousness on occupational commitment mediated by just culture

Every factor loading was statistically significant (CR > 1.96, *p* < 0.001), indicating that the higher-order constructs had a strong ability to predict their subdimensions. Occupational commitment was substantially associated with affective, normative, alternative cost, and alternatives, whereas organizational virtuousness significantly predicted optimism, trust, compassion, integrity, and forgiveness. Likewise, transparency, balance, feedback and communication, event-reporting quality, and ongoing development were all highly predicted by just culture. All things considered, the model showed a good to adequate fit (Table 6; Fig. 3).

## Discussion

In this study, the participants’ average age was 41.9 years (SD = 7.80), suggesting that the nursing workforce is comparatively experienced and mature. Regarding to the gender distribution shows a significant female majority (77.3%), which is in line with the nursing profession’s historically female-dominated character around the world. Additionally, the fact that 47.8% of the participants were married may help us understand their support networks, stress levels, and possible work-life conflict factors that are frequently associated with occupational commitment and job satisfaction. Furthermore, 64% of the study participants had more than 15 years, which leads to the experienced nurses tending to be more introspective and knowledgeable about organizational culture and attitudes connected to their jobs. This degree of professional longevity lends credence to responses.

### Distribution of the levels and mean percent score of organizational virtuousness, just culture, and occupational commitment among nurses

Moderate-to-high levels of organizational virtuousness have been detected, indicating a generally positive perception of the moral and ethical atmosphere at work. Furthermore, forgiveness (65.02%) ranked first, suggesting that the healthcare organization culture probably encourages second chances and learning from errors. These results aligned with Bashandy et al. (2024), which revealed that almost three-quarters of nurses gave “high” ratings for the overall organizational virtuousness level. According to the results, most of the nurses in the study gave “high” answers to the “meaning” dimension, which was followed by the percentage who gave “high” answers to the “forgiveness” component [34].

Additionally, 71.8% (*n* = 287) of respondents expressed high levels of occupational commitment. This indicates a deep emotional and social bond with the field. Additionally, significantly high levels of normative commitment (70.50%) and emotional commitment (69.07%) suggest that nurses have a strong sense of moral obligation and emotional investment in their work. This study aligns with Elkady et al. (2021), which revealed that the mean scores of nurses at governmental and non-governmental hospitals regarding overall organizational commitment differed in a very significant way [35].

Furthermore, the strongest construct was just culture, as indicated by the high scores of 84.8% (*n* = 339) of nurses, which means expressing a favorable opinion of the organization’s impartiality and commitment to learning. All subscales were led by Continuous Improvement (71.17%), indicating a proactive culture that prioritizes learning and improvement. On the same line, Logroño et al. showed that the just culture had an overall good perception score of 75.44%. The “continuous improvement” feature was one of Just Culture’s strong points [36].

### Correlation between organizational virtuousness, just culture, and occupational commitment among nurses

This study revealed a weak but statistically significant positive association between Just Culture and Organizational Virtuosity that supports hypothesis (1), which, due to the existence of a fair culture, is positively correlated with judgments of organizational virtuousness, which are characterized by honesty, trust, compassion, forgiveness, and optimism. This association has theoretical significance despite its weakness. This research supports the idea that morally and ethically driven leadership practices foster an environment where learning from mistakes, fairness, and transparency are accepted as standard operating procedures. Furthermore, Organizational virtuousness and occupational commitment which support hypothesis (2), have a weak but significant positive association, suggesting that nurses who believe their organizations uphold moral principles are also more likely to experience a strong sense of dedication to their work. Despite its weakness, this association highlights the indirect motivational effect of a values-driven workplace on commitment to one’s work. A sense of purpose and congruence with personal values can be fostered by an organization’s virtue, which can increase emotional and normative commitment (Meyer & Allen, 1991). This demonstrates how company culture helps to strengthen employees’ sense of self, pride, and loyalty [13].

Additionally, A more developed just culture is linked to better occupational commitment among nurses. Nurses are more likely to feel safe, appreciated, and committed when their organizations promote justice, accountability, transparency, and ongoing learning. This is consistent with research showing that a just culture improves engagement, morale, and organizational trust [37]. Additionally, data suggests that nurses are more dedicated when they feel comfortable discussing mistakes, reporting occurrences, and feeling that their efforts are appropriately evaluated.

### Linear regression models for factors affecting occupational commitment

Both organizational virtuousness and just culture considerably contribute to explaining the variance in occupational commitment, according to the results of the linear regression analysis. About 15.5% of the variation in occupational commitment was explained by the whole model, which was statistically significant. According to these results, culture alone has a greater impact on professional commitment, even though both factors are significant. To improve nurses’ involvement and long-term commitment to their profession, it is imperative to create a work atmosphere that is equitable, encouraging, and learning oriented. This result is consistent with Kim et al. (2017), which highlights that improving occupational commitment and retention requires organizational justice and moral treatment of workers (empowerment, support, and moral leadership [38].

### SEM for the relationship between organizational virtuousness, just culture, and occupational commitment among nurses

All observable indicators loaded heavily on their respective latent constructs, according to the results of structural equation modeling, confirming the validity of the measurement model. Its subdimensions were significantly predicted by organizational virtuousness, with integrity showing the highest standardized loading, followed by forgiveness, compassion, and trust. Likewise, occupational commitment was well represented by its dimensions: the highest factor loading was found for alternative cost, which was followed by normative commitment, affective commitment, and alternatives. All the subdimensions strongly loaded into the latent variable regarding just culture: balance, feedback and communication, quality of event reporting procedure, openness of communication, and continuous improvement exhibited the strongest estimate. In the context of organizational virtuousness, just culture, and occupational commitment, these findings support the measurement model’s resilience and show that each dimension is a reliable indicator of its corresponding construct, and just culture plays a mediating role between organizational virtuousness and nurses’ occupational commitment; these results support hypothesis (4).

## Conclusion

This study offers empirical proof that organizational virtue, both directly and indirectly through the mediation function of fair culture, greatly increases nurses’ vocational commitment. When nurses believe their organization is moral, encouraging, and equitable, they exhibit greater emotional and normative commitment. A key tool for converting corporate principles into significant employee involvement is just culture. These results emphasize how crucial it is to create ethical workplaces that value fairness, candid communication, and ongoing development to increase stability and loyalty in the healthcare industry. The study provides insightful information for nursing organizations’ leadership practices and policy improvement.

### Strengths and limitations

This study’s theoretical contribution, which combines organizational virtue and just culture into a coherent model to predict occupational commitment, is one of its main strengths. The findings’ reliability and generalizability in comparable healthcare settings are increased using validated instruments, a large sample selected from many critical care units, and a strong statistical methodology using structural equation modeling (SEM). Furthermore, the emphasis on a public university hospital in Egypt adds contextual relevance for low and middle-income countries (LMICs), where organizational culture improvements are essential. The study does have several drawbacks, though. Self-reported data may be prone to response bias, and its cross-sectional design restricts the ability to conclude causality. Moreover, the results could not apply to non-academic or private healthcare facilities. It is advised that these findings be confirmed and expanded upon in future studies employing mixed methods or longitudinal approaches.

### Implications of the study

#### Implications for nursing practice

The human-centered approach and moral commitment to patient care make the nursing profession unique. To empower nurses, nursing leaders should cultivate a culture of communication, support, and ongoing education. Nurses’ career maturity and patient outcomes are enhanced when active organizational learning is promoted [39]. Organizational intelligence has been demonstrated to foster soft skills and thriving among nurses, highlighting the fundamental importance that supportive organizational environments play in improving nurses’ abilities and well-being (Atalla et al., 2024). These results support the necessity for psychologically secure and learning-oriented workplaces by highlighting Just Culture as a mediation mechanism via which organizational virtues translate into increased occupational commitment [40]. Building on earlier research, Atalla et al. (2025) showed that nurses’ proficiency in evidence-based practice and organizational enablers can be mediated by promoting self-efficacy. By enabling nurses to behave confidently and morally in their workplaces, this finding bolsters the current study’s hypothesis that internal mediators, like Just Culture, are essential in converting organizational virtues into favorable professional outcomes like occupational commitment [41].

The findings highlight how crucial it is to foster an equitable and morally sound work environment to increase nurses’ dedication to their careers. When nurses believe their organization is fair, encouraging, and morally upright, they are more likely to stay involved and committed, even in difficult clinical environments. Integrating integrity, compassion, and respect into nursing practice daily promotes moral alignment and a sense of belonging, which in turn improves job satisfaction and lowers turnover intentions. Nurses are further empowered to actively contribute to the creation of just and moral care environments by promoting reflective practice, peer support, and open communication.

#### Implications for nursing management

This study highlights the essential role of leadership in modeling and progressing organizational virtue and a just culture within nursing management. By emphasizing openness in decision-making, guaranteeing equitable treatment, and taking non-punitive stances when mistakes are made, nurse managers can promote psychological safety and trust. In addition to upgrading team cohesion, nurses’ professional dedication can be further increased by establishing a learning-oriented culture and putting in place organized feedback mechanisms. Leadership development programs should incorporate conflict resolution, emotional intelligence, and ethical leadership training to equip managers to lead with compassion and integrity.

#### Implications for nursing education and policy

To equip future nurses to deal with ethical and organizational encounters, nursing curricula should integrate concepts of organizational virtuousness and just culture. Educational programs can incorporate case-based learning and simulation scenarios that emphasize justice, equity, and accountability in complex healthcare settings. At the policy level, healthcare organizations and regulatory bodies should embed just culture principles into national nursing standards and certification frameworks. Furthermore, uniform policies that promote equity, continuous education, and ethical practice will not only strengthen workplace culture but also ensure sustainable professional growth.

#### Implications for future research

Future research on the dynamic interaction between organizational culture and nursing outcomes in many healthcare environments is made possible by this study. To investigate how long-term exposure to moral settings and just cultural practices affects nurses’ career paths and long-term commitment, longitudinal and mixed-method research is required. Future studies should also investigate how institutional, cultural, and demographic factors may moderate these associations, especially in low- and middle-income nations. Context-specific therapies and the generalization of findings would both benefit from comparative research conducted across various healthcare systems.

## Supplementary Information

Below is the link to the electronic supplementary material.

Supplementary Material 1

## Data Availability

Upon reasonable request, the corresponding author will provide the datasets created and examined during the current work.
