# Supplementary material for: Exploring how organizational virtuousness shapes nurses’ occupational commitment: the mediating role of just culture
Source: BMC Nurs. 2025 Sep 10;24:1167. doi: 10.1186/s12912-025-03835-x (PMC12424223; doi:10.1186/s12912-025-03835-x)
Supplement: Supplementary file 1 — Supplementary Material 1 [file 12912_2025_3835_MOESM1_ESM.docx]

**Supplementary Table 1. Correlations among subscales of Organizational Virtuousness, Just Culture, and Occupational Commitment (n = 400)**

**Section A. Organizational Virtuousness Subscales**

| **Variable** | **1. Optimism** | **2. Trust** | **3. Compassion** | **4. Integrity** | **5. Forgiveness** | **6. OV (Total)** |
| --- | --- | --- | --- | --- | --- | --- |
| 1. Optimism | 1 |  |  |  |  |  |
| 2. Trust | 0.642* | 1 |  |  |  |  |
| 3. Compassion | 0.749* | 0.636* | 1 |  |  |  |
| 4. Integrity | 0.663* | 0.740* | 0.767* | 1 |  |  |
| 5. Forgiveness | 0.641* | 0.583* | 0.686* | 0.653* | 1 |  |
| 6. OV (Total) | 0.853* | 0.846* | 0.891* | 0.893* | 0.821* | 1 |

**Section B. Occupational Commitment Subscales**

| **Variable** | **1. Affective** | **2. Normative** | **3. Alternative Cost** | **4. Alternatives** | **5. Continuance** | **6. OC (Total)** |
| --- | --- | --- | --- | --- | --- | --- |
| 1. Affective | 1 |  |  |  |  |  |
| 2. Normative | 0.690* | 1 |  |  |  |  |
| 3. Alt. Cost | 0.574* | 0.565* | 1 |  |  |  |
| 4. Alternatives | 0.671* | 0.629* | 0.642* | 1 |  |  |
| 5. Continuance | 0.650* | 0.630* | 0.972* | 0.804* | 1 |  |
| 6. OC (Total) | 0.850* | 0.843* | 0.864* | 0.820* | 0.921* | 1 |

**Section C. Just Culture Subscales**

| **Variable** | **1. Feedback & Comm.** | **2. Openness** | **3. Balance** | **4. Event Reporting** | **5. Continuous Improvement** | **6. JC (Total)** |
| --- | --- | --- | --- | --- | --- | --- |
| 1. Feedback & Comm. | 1 |  |  |  |  |  |
| 2. Openness | 0.672* | 1 |  |  |  |  |
| 3. Balance | 0.652* | 0.609* | 1 |  |  |  |
| 4. Event Reporting | 0.462* | 0.494* | 0.581* | 1 |  |  |
| 5. Continuous Improvement | 0.538* | 0.568* | 0.651* | 0.587* | 1 |  |
| 6. JC (Total) | 0.816* | 0.846* | 0.859* | 0.774* | 0.762* | 1 |
